# Integrity in Radiation Oncology Research: A Systematic Review of Retracted Studies, Retraction Notices, and Their Impact on the Field

**DOI:** 10.1016/j.adro.2026.102071

**Published:** 2026-05-01

**Authors:** Philip Heesen, Maksym Fritsak, Lena Kretzschmar, Astrid Heusel, Olga Ciobanu-Caraus, Maiwand Ahmadsei, Maximilian Thormann, Felix Ehret, Nicolaus Andratschke, Daniel Zips, Matthias Guckenberger, Siyer Roohani, Sebastian M. Christ

**Affiliations:** a Department of Radiation Oncology, University Hospital Zurich and University of Zurich, Zurich, Switzerland; b Faculty of Medicine, University of Zurich, Zurich, Switzerland; c Department of Neurosurgery, LMU Hospital Munich, Munich, Germany; d Charité – Universitätsmedizin Berlin, Corporate Member of Freie Universität Berlin and Humboldt-Universität zu Berlin, Department of Nuclear Medicine, Berlin, Germany; e Charité - Universitätsmedizin Berlin, Corporate Member of Freie Universität Berlin and Humboldt-Universität zu Berlin, Department of Radiation Oncology, Berlin, Germany; f German Cancer Consortium (DKTK), partner site Berlin, a partnership between DKFZ and Charité - Universitätsmedizin Berlin, Germany, Heidelberg, Germany; g Radiation Medicine Program, Princess Margaret Cancer Centre, Department of Radiation Oncology, University of Toronto, University Health Network, Toronto, Ontario, Canada; h BIH Biomedical Innovation Academy, BIH Charité (Junior) Clinician Scientist Program, Berlin Institute of Health der Charité - Universitätsmedizin Berlin, Berlin, Germany

## Abstract

**Purpose:**

Retractions in the scientific literature undermine research integrity and can have lasting effects on clinical and academic practices. The aim of this systematic review was to analyze retracted publications in the recent radiation oncology literature and to identify trends in study characteristics, reasons for retraction, and postretraction citation patterns.

**Methods and Materials:**

A systematic review was conducted following the Preferred Reporting Items for Systematic Reviews and Meta-Analyses guidelines. Retracted studies in radiation oncology were identified from PubMed, Embase, Cochrane Central, and Retraction Watch, from May 1, 2017, to September 27, 2024, and June 16, 2025, respectively. Studies were included if they had a publicly available retraction notice. Two independent reviewers screened studies and extracted data on study characteristics, retraction reasons, and postretraction citation patterns. Descriptive statistics were used for data synthesis.

**Results:**

A total of 108 retracted studies were identified and analyzed. Most retracted studies were laboratory/experimental studies (39.8%). Median impact factor of retracted studies was 2.4 (IQR, 1.8-3.5). The primary reason for retraction was misconduct (77.8%), followed by methodological errors (13.9%) and authorship issues (5.6%). Retraction notices were issued primarily by publishers (50.0%). Most retracted articles (93.5%) were marked with a watermark, and retractions were indexed in PubMed in all cases. Median time from publication to retraction was 1.6 years (IQR, 1.0-3.2 years). Median number of citations after retraction was 1 (IQR, 0-3).

**Conclusions:**

This study found that retractions in radiation oncology remain generally rather rare events. However, prevalence of misconduct-related retractions in radiation oncology is high, and citations may persist after retraction. These findings emphasize the need for further enhanced oversight and clearer retraction policies to foster high-quality and sound research practices.

## Introduction

In academic publishing, a retraction is a process used to flag a published paper for containing serious flaws, making its results and conclusions unreliable. Retractions are an important mechanism for preserving the accuracy and credibility of research. Even though retractions are a relatively rare event, they have become more frequent.^1^^,^^2^ Retractions typically imply serious concerns, such as methodological flaws, ethical breaches, or falsified data, which have come to light after publication. The retraction of a single study rarely leads to immediate shifts in clinical practice, but it can trigger broader scrutiny of the evidence base, drive improvements in research methodology, and help strengthen the foundations of patient care over time.^3^ Hence, by tracking and better understanding patterns and trends in retractions, the scientific community can ensure that research remains a reliable foundation for decision-making.

Wasiak et al^4^ published a survey of retracted studies in radiation oncology in 2018, which explored the trends and causes of retractions within the field, offering a comprehensive analysis of the period leading up to 2017. This study found that retractions in radiation oncology are rather rare events, yet retracted studies often continue to be cited in the literature.^4^ Hamilton^5^ further substantiated this analysis by assessing the frequency of the postretraction citations of these articles, finding that 92% of postretraction citations referenced retraced articles as legitimate work. Since the publication of Wasiak et al’s^4^ analysis, no subsequent research has been conducted to update these findings. This represents a notable gap in our understanding of how retraction trends have evolved over the past several years in our discipline.

Over the past decade, the number of scientific publications has experienced a steady and significant increase.^6^ This growth is particularly evident in fields like radiation oncology, which have seen an influx of research addressing a wide array of emerging technologies, treatment approaches, and diagnostic tools.^7^ Although the increase in publication output is generally seen as a positive sign of scientific advancement, it has also raised concerns regarding the overall quality and reproducibility of research.^8^ The rapid publication pace, combined with pressures to publish in high-impact journals, has at times contributed to compromised research practices, such as inadequate peer review processes, data manipulation, and statistical errors.^9^ As a result, the number of retracted articles has been on the rise across many scientific disciplines, including radiation oncology, medical imaging, and medical physics.^10^, ^11^, ^12^, ^13^, ^14^, ^15^, ^16^

Additionally, the COVID-19 pandemic from 2020 to 2022 has brought about significant disruptions to the academic and scientific communities. The urgency to respond to the public health crisis has led to shifts in research priorities, with an increased focus on topics directly related to the pandemic, including cancer care adjustments during the crisis and the development of new treatment protocols. The pandemic has also had an impact on research funding, collaboration, and access to resources, which in turn may have affected the rigor and oversight of studies published during this period.^17^ It remains unclear whether the COVID-19 pandemic has exacerbated or alleviated retraction trends in radiation oncology or if it has introduced new dynamics that warrant further investigation.^18^

Given these factors, it is both timely and necessary to revisit the issue of retractions in radiation oncology. This systematic review builds upon the work of Wasiak et al^4^ by leveraging more recent and comprehensive data, and by shifting the timeframe of analysis to publications after 2017. This updated analysis considers the impact of COVID-19 on research practices and publication trends. It aims to clarify current retraction trends in radiation oncology, highlight emerging causes, and suggest ways to prevent future issues in academic publishing in our field.

## Methods and Materials

### Protocol and registration

This systematic review was conducted following the Preferred Reporting Items for Systematic Reviews and Meta-Analyses guidelines.^19^ (Appendix E1) given that the research was based exclusively on publicly available data, no institutional review board approval was required.

### Eligibility criteria

To ensure a comprehensive assessment of retractions, we applied the following inclusion and exclusion criteria: we considered studies that (1) were published in peer-reviewed journals and referenced any aspect of radiation oncology, including, but not limited to radiation therapy planning, treatment methodologies, radiation biology, and medical physics and (2) had been formally retracted, with an accompanying publicly available retraction notice explaining the reason for removal from the literature. Studies were excluded if (1) full-text access was unavailable, preventing a thorough evaluation of the content; (2) the publication language was not English, as translation inconsistencies could have introduced biases; and (3) the article was marked as an “expression of concern” without full retraction, as these do not indicate a definitive retraction from the scientific record. We placed no restrictions on the type of study design (eg, randomized controlled trials, observational studies, reviews, case reports) or publication type, ensuring a broad and comprehensive data set.

### Information sources and search strategy

First, we conducted a systematic literature search across 3 major electronic databases: PubMed, Embase, and Cochrane Central. The search terms were designed to capture all relevant retractions within radiation oncology, as defined and employed in similar studies.^20^ Details regarding search strategy and search strings can be found in Appendix E2. The search covered studies published from May 1, 2017, to September 27, 2024, ensuring an up-to-date assessment of recent trends in retractions. The search was performed on September 27, 2024. Secondly, the Retraction Watch database was searched on June 16th, 2025, in order to complement and cross-check studies identified in the systematic literature search against this publicly available resource. Additionally, manual searches of reference lists from key articles were performed to identify any missed studies.

### Selection process

To enhance efficiency and reduce reviewer burden, we first applied a rule‑based text‐processing and pattern‑based data‐extraction pipeline to prescreen titles, flagging potentially relevant studies. For both PubMed and Embase, we ran a custom Python script to isolate truly retracted papers, and excluded records were manually reviewed to guard against false negatives. Confirmed retractions were then screened for “radiotherapy relevance” using a curated list of over 40 radiation therapy–related terms, originally developed for clinical trial screening.^20^ Retraction Watch data, because of its smaller size, was processed in semiautomatic way, by verifying whether each retraction is related to the radiation oncology field and not listed in PubMed and Embase data sets. We have made all preprocessing scripts and resulting data sets publicly available on GitHub (see Appendix E2 for URL). Following prescreening, studies were imported into Rayyan, a web-based platform designed to streamline systematic review processes (https://www.rayyan.ai/). In a first iteration, 2 independent reviewers conducted a thorough full-text screening of all identified studies; in cases of disagreement, a third senior reviewer was consulted to reach a consensus. In a second iteration, 2 other research team members reviewed the results of the full-text screening process for quality assurance purposes.

### Data collection process

Data extraction was performed using a standardized data extraction form, which was developed to ensure consistency across all reviewed studies. The form was developed a priori by 2 members of the research team based on the study objectives and informed by data extraction instruments used in similar retraction analyses.^4^^,^^14^ It was pilot-tested on a subset of 5 retracted studies to assess completeness and usability, and subsequently refined before full-scale extraction. Two independent reviewers extracted key study characteristics, including publication details (authors, journal, year of publication, and country of origin), retraction specifics (date of retraction and presence of retraction notice), reasons for retraction (categorized into misconduct, methodological errors, or authorship disputes), study type (eg, clinical trial, observational study, or review), and citation impact (if available, for 2024, to assess whether the study was cited postretraction). Discrepancies in extracted data were resolved through discussion and, if necessary, adjudicated by a third reviewer. During the manual data extraction phase, we applied the following methods to guarantee internal consistency of all data points:

1. The date of first online availability of the publication was used as the publication date because we considered electronic publication to be the form in which articles are generally most quickly and widely distributed. Print dates were therefore not taken into account.
2. The country of origin of the respective article was defined as the location of the institution of the first author’s affiliation.
3. Retracted articles were categorized into preretraction and postretraction citations. Postretraction citations were defined as citations that refer to the respective article in its intended function and use the information provided in it as scientific evidence. More concretely, this means that any metatextual citations, eg, retraction notes, letters to the editor, or articles that refer to retracted papers postretraction to gather scientific evidence not about the subject matters of the publications themselves, but about scientific misconduct or other methodical problems associated with them, were not included in the postretraction citations count. Additionally, postretraction citations were only marked as such if the latency time between the online publication date of the retraction note and the online publication date of the citing paper exceeded a 1-month grace period. If the latency was shorter than 1 month, we assumed that—this is close to the publication of a scientific article—no reaction to the retraction note, ie, no correction/deletion of the citation would be possible anymore, and therefore included any article that fell into this grace period as a preretraction citation.

### Study risk of bias assessment

Because this review focused on retracted studies rather than the methodological quality of original research, a formal risk of bias assessment was not conducted. However, to ensure the accuracy of extracted data and minimize misclassification errors, 2 reviewers independently verified retraction details against official retraction notices published by journals or indexing databases. Additionally, we assessed whether retraction notices provided sufficient detail about the reasons for retraction, as vague or missing explanations could impact our categorization of retraction causes.

### Data synthesis and analysis

To summarize the characteristics and trends of retracted studies, we performed a descriptive statistical analysis. The following measures were used: frequencies and percentages for categorical variables (eg, reasons for retraction and study types) and medians and IQRs for continuous variables (eg, time from publication to retraction). The reasons for retraction were categorized into 3 main groups: (1) misconduct, including plagiarism, data fabrication, falsification, peer review manipulations, or ethical violations; (2) methodological errors, such as analytical flaws, incorrect study design, or reproducibility concerns; and (3) authorship issues, including disputes over contributor roles or failure to meet journal authorship criteria. Although at the time of revision of our data, we attempted to further subdivide misconduct into the subcategories listed above to allow for a more detailed analysis, we found that a significant number of papers fit into more than one of these categories, severely limiting the usability of the subcategories, which is why we eventually decided on keeping the umbrella term misconduct as unifying category to most adequately represent the reason for retraction. Moreover, studies were categorized as published before the COVID-19 pandemic if they were published before November 17, 2019—the day the first case is believed to have occurred in Wuhan, China, according to Chinese government records.^21^ All statistical analyses were conducted using R Project (version 4.4.1).

## Results

**Figure 1:**
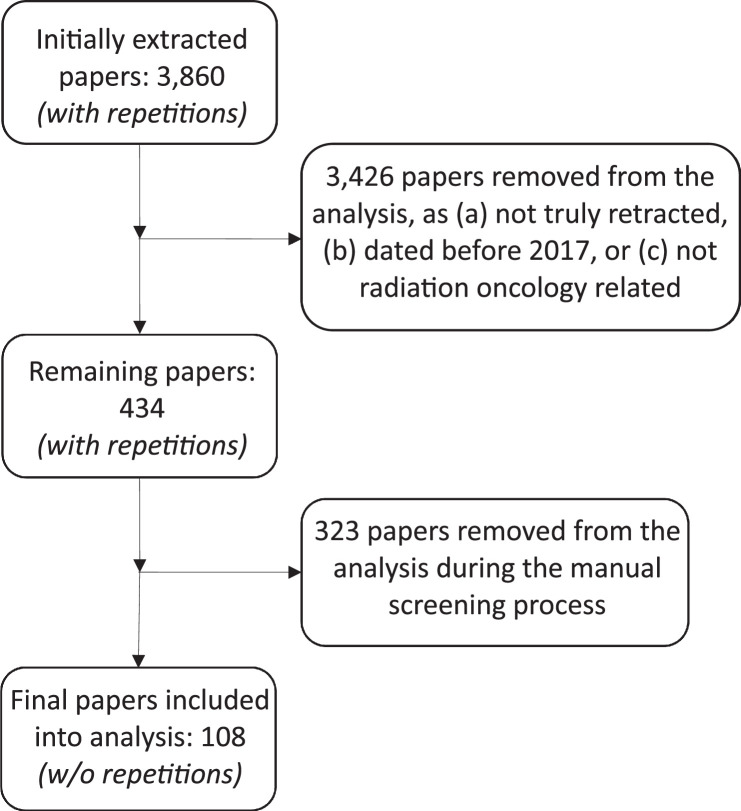
Preferred Reporting Items for Systematic Reviews and Meta-Analyses flow diagram of study.

Across all data sources used, a total of 3860 records were initially identified, of which 108 (2.8%) were included in the analysis (Appendix E3). The inclusion process is presented in Fig. 1. The median impact factor of the journals in which these studies were published was 3.1 (IQR, 2.7-3.9; range, 0.5-35.9). The journals with the most retractions were *Journal of Healthcare Engineering* (14/108; 13.0%), followed by *Evidence-based Complementary and Alternative Medicine* (11/108; 10.2%) and *Computational and Mathematical Models in Medicine* (8 of 63; 7.4%). The journals with the highest impact factor were *Lancet Oncology* (1 of 108; 0.9%) and *Cancer Research* (1/108; 0.9%). The median number of authors per study was 6 (IQR, 4-7; range, 1-21). The majority of the analyzed studies (75 of 108; 69.4%) originated from China. Other repeatedly represented countries included the United States (11 of 10.2; 14.8%), Iran (4 of 108; 3.7%), and Canada (3 of 108; 2.8%). Turkey, Mexico, Greece, and South Korea each accounted for 2 studies (2 of 108; 1.9%). Saudi Arabia, Morocco, Bangladesh, Italy, India, Germany, and France accounted for 1 study each (1 of 108; 0.9%).

Among study designs, laboratory/experimental studies made up the largest proportion (43 of 108; 39.8%), followed by retrospective observational studies (29 of 108; 26.9%). Prospective observational and prospective interventional studies accounted for 11 studies each (10.2%).

Systematic reviews/meta-analyses comprised 5.6% (6 of 108), while case reports comprised 4.6% (5 of 108). Randomized trials constituted 2.8% (3 of 108) of retracted studies. The most frequently cited reason for retraction was misconduct, which was reported in 77.8% (84 of 108) of cases. Out of those, 43 studies were retracted because of systematic peer review manipulation and methodological inconsistencies (43 of 84; 51.2%). Methodological errors accounted for 13.9% (15/108) of retractions, while authorship issues were identified in 5.6% (6 of 108). One study (1 of 108, 0.9%) was withdrawn from the Cochrane Library because the review was out of date and redundant. For 2 studies, the reason for retraction was unclear (2 of 108, 1.8%). Retraction notices were issued most often by publishers (54 of 108, 50.0%), with editors and authors issuing 23.1% (25 of 108) and 20.4% (22 of 108) of notices, respectively. In 3.7% (4 of 108) of cases, both the author and editor were listed as issuing the retraction, while in 2.8% (3 of 108), the publisher and editor issued the retraction. Table 1 summarizes key variables.

**Table 1:** Characteristics of included studies and retraction details

Variable	N = 108
Country of the first author	
China	75 (69.4%)
United States	11 (10.2%)
Iran	4 (3.7%)
Canada	3 (2.8%)
Greece	2 (1.9%)
Mexico	2 (1.9%)
South Korea	2 (1.9%)
Turkey	2 (1.9%)
Bangladesh	1 (0.9%)
France	1 (0.9%)
Germany	1 (0.9%)
India	1 (0.9%)
Italy	1 (0.9%)
Morocco	1 (0.9%)
Saudi Arabia	1 (0.9%)
Number of authors	6 (4, 7)
Impact factor (year 2024)	3.1 (2.7, 3.9)
Study design	
Laboratory/experimental	43 (39.8%)
Retrospective observational	29 (26.9%)
Prospective observational	11 (10.2%)
Prospective interventional	11 (10.2%)
Systematic review/meta-analysis	6 (5.6%)
Case report	5 (4.6%)
Randomized controlled trial	3 (2.8%)
Number of citations before retraction	0.0 (0.0, 5.0)
Reason for retraction	
Misconduct	84 (77.8%)
Methodological error	15 (13.9%)
Authorship issue	6 (5.6%)
Out of date review	1 (0.9%)
Unclear	2 (1.8%)
Issuer of retraction note	
Publisher	54 (50.0%)
Editor	25 (23.1%)
Author	22 (20.4%)
Author and editor	4 (3.7%)
Publisher and editor	3 (2.8%)
Indication of retraction	
Watermark	101 (93.5%)
None	4 (3.7%)
In-text notation	3 (2.8%)
Number of citations after retraction	1 (0, 3)
Retraction notification in PubMed	104 (96.3%)
Time from publication to retraction (y)	1.6 (1.0, 3.2)

Numbers are presented as n (%) or median (IQR).

**Figure 2:**
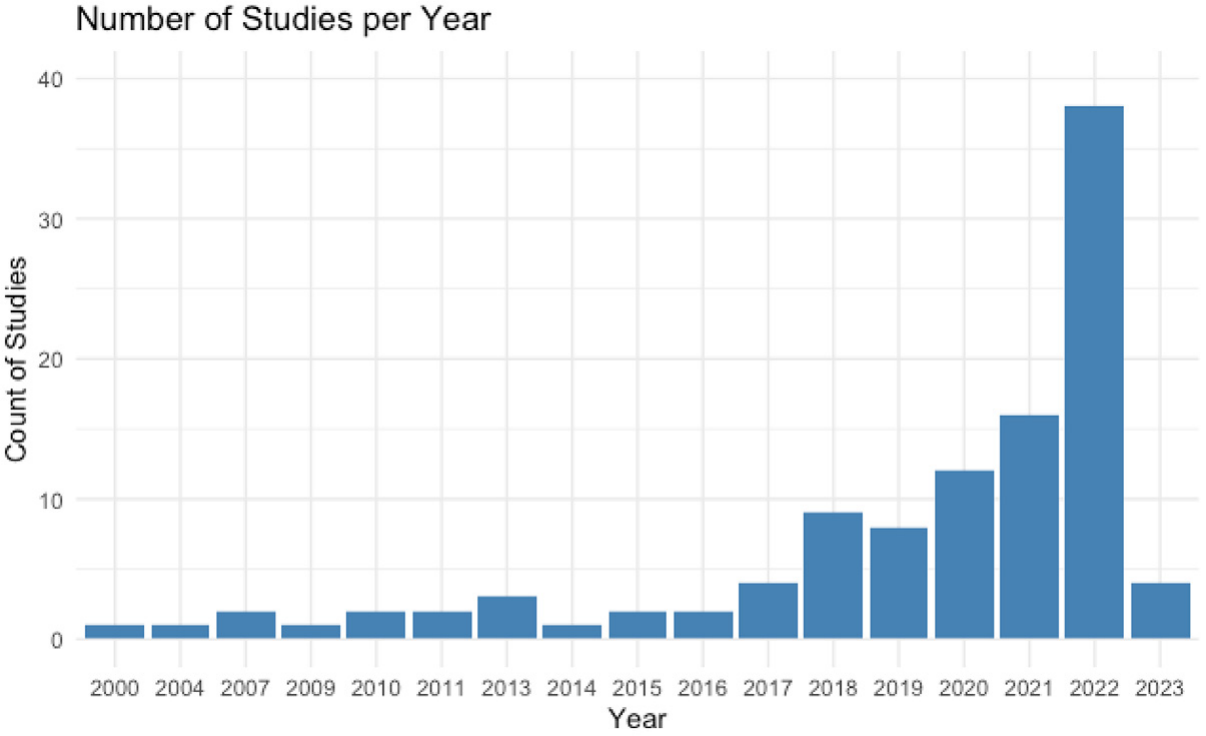
Bar chart of the number of studies retracted by their year of publication.

A majority of 93.5% (101 of 108) retracted studies were marked by a watermark, 3.7% (4 of 108) had no indication of retraction, and 2.8% (3 of 108) included an in-text notation. The median number of citations before retraction was 1 (IQR, 0-5; range, 0-97), while the median number of citations after retraction was 1 (IQR, 0-3; range, 0-18). Retractions were recorded in PubMed in 96.3% (104 of 108) of cases, whereas in 4 cases (2.7%) they were not*.* The median time from publication to retraction was 1.6 years (IQR, 1.0-3.2; range, 0-0-17.3 years). Most retracted studies were initially published in the year 2022 (38 of 108; 35.2%), followed by the year 2021 (16 of 108, 14.8%). Figure 2 presents the number of retracted studies by publication year. Thirty studies (30 of 108; 27.8%) were published before the start of the COVID-19 pandemic, whereas 78 (78 of 108; 72.2%) studies were published during and after the COVID-19 pandemic. Table 2 displays the study characteristics and retraction details stratified by publication date before or after the beginning of the COVID-19 pandemic.

**Table 2:** Characteristics of studies and retraction details stratified by time period before or after COVID-19

Variable	Before COVID-19 (n = 30)	After COVID-19 (n = 78)
Country		
Bangladesh	0 (0.0%)	1 (1.3%)
Canada	2 (6.7%)	1 (1.3%)
China	11 (36.7%)	64 (82.1%)
France	1 (3.3%)	0 (0.0%)
Germany	1 (3.3%)	0 (0.0%)
Greece	2 (6.7%)	0 (0.0%)
India	0 (0.0%)	1 (1.3%)
Iran	1 (3.3%)	3 (3.8%)
Italy	0 (0.0%)	1 (1.3%)
Mexico	1 (3.3%)	1 (1.3%)
Morocco	1 (3.3%)	0 (0.0%)
Saudi Arabia	1 (3.3%)	0 (0.0%)
South Korea	1 (3.3%)	1 (1.3%)
Turkey	0 (0.0%)	2 (2.6%)
USA	8 (26.7%)	3 (3.8%)
Number of authors	7 (6, 8)	5 (3, 7)
Impact factor (year 2024)	3.2 (2.1, 4.5)	3.0 (2.7, 3.8)
Study design		
Case report	3 (10.0%)	2 (2.6%)
Laboratory/experimental	20 (66.7%)	23 (29.5%)
Prospective interventional	1 (3.3%)	10 (12.8%)
Prospective observational	4 (13.3%)	7 (9.0%)
Randomized controlled trial	0 (0.0%)	3 (3.8%)
Retrospective observational	1 (3.3%)	28 (35.9%)
Systematic review/meta-analysis	1 (3.3%)	5 (6.4%)
Number of citations before retraction	4 (2, 11)	1 (0, 3)
Reason for retraction		
Misconduct	21 (70.0%)	63 (81.8%)
Methodological error	6 (20.0%)	9 (11.7%)
Authorship issue	1 (3.3%)	5 (6.5%)
Review out of date	1 (3.3%)	0 (0.0%)
Unclear	1 (3.3%)	1 (1.3%)
Issuer of retraction note		
Publisher	3 (10.0%)	51 (65.4%)
Editor	11 (36.7%)	14 (17.9%)
Author	11 (36.7%)	11 (14.1%)
Author and editor	3 (10.0%)	1 (1.3%)
Publisher and editor	2 (6.7%)	1 (1.4%)
Indication of retraction		
Watermark	27 (90.0%)	74 (94.9%)
None	2 (6.7%)	2 (2.6%)
In-text notation	1 (3.0%)	2 (2.6%)
Number of citations after retraction	1 (0, 3)	1 (0, 2)
Retraction notification in PubMed	28 (93.3%)	76 (98.7%)
Time from publication to retraction (y)	5.5 (3.1, 10.7)	1.3 (0.9, 2.0)

Numbers are presented as n (%) or median (IQR).

To explore whether retracted studies shared common thematic elements, we categorized each study by research theme based on its title (categories were not mutually exclusive). The most prevalent category was molecular biology and signaling pathway research, including studies on microRNAs, long noncoding RNAs, and gene-level mechanisms of radiosensitization (31 of 108, 28.7%), followed by combined modality therapy evaluating chemotherapy or immunotherapy in combination with radiation therapy (29 of 108, 26.9%), and treatment planning, dosimetry, or medical physics (20 of 108, 18.5%). Studies addressing clinical outcomes or prognostic factors and radiation toxicity or side effects management each accounted for 15.7% (17 of 108). Natural products or novel agents investigated as radiosensitizers comprised 12.0% (13 of 108), while studies involving artificial intelligence (AI), computational methods, or radiomics accounted for 9.3% (10 of 108). Studies on radiosensitization or radioresistance mechanisms comprised 8.3% (9 of 108), imaging-based biomarker studies 6.5% (7 of 108), and nursing, supportive care, or quality of life studies 2.8% (3 of 108).

## Discussion

In this analysis, the dominance of studies originating from China (69.4%) is notable, yet this was also the case in similar recent assessments, for example, of the medical imaging literature.^14^ This finding also aligns with a growing body of literature that highlights China’s rapidly expanding research output.^22^ Research from China has been subject to scrutiny in recent years because of concerns on data fabrication and authorship issues.^23^, ^24^, ^25^ However, this finding is not intended to reflect on any country or its research community as a whole. Scientific integrity and adherence to publication ethics are universal responsibilities that apply to all researchers, regardless of geographic origin. Encouragingly, efforts to strengthen research integrity are ongoing, including recent proposals for improvement in China.^26^^,^^27^ Our goal is to support and strengthen ethical standards in radiation oncology research worldwide. This high percentage of Chinese studies may also reflect broader trends in research production and potential issues related to academic misconduct in rapidly developing research environments.^28^ The smaller contributions from other countries align with findings in other studies, although some studies have noted the importance of cross-national differences in retraction rates.^28^ In the analysis by Wasiak et al,^4^ which assessed studies up until 2017, 34% of studies emanated from Asia and Europe, respectively. In the future, it may be useful to consider how cultural or institutional factors in different countries might influence the occurrence and detection of retractions.

Regarding the reasons for retraction, misconduct (77.8%) was the reason for retraction in the majority of cases in this analysis. This finding is consistent with existing research, which has found that a substantial proportion of retracted studies are because of scientific misconduct, including data fabrication, falsification, and plagiarism.^10^, ^11^, ^12^, ^13^ In fact, the prevalence of misconduct as a primary cause of retraction has been well-documented, and it suggests the ongoing challenges in maintaining the integrity of the scientific process.^29^, ^30^, ^31^, ^32^ Although methodological errors (13.9%) and authorship issues (5.6%) were also detected in this analysis, they are less frequent, yet contribute to retraction events, which is also in line with comparable research studies.^14^^,^^29^, ^30^, ^31^, ^32^ This study, hence, confirms the top 3 reasons for retraction in the recent radiation oncology literature previously reported by Wasiak et al,^4^ for the period leading up to 2017.

Scientific misconduct in radiation oncology, as highlighted by the high proportion of retractions due to fraud (77.8%), can be interpreted as a symptom of systemic flaws in academia, driven largely by a “publish or perish” culture. The pressure to secure funding and career advancement through high publication counts fosters an environment where integrity can be compromised, leading to data fabrication, plagiarism, and peer review manipulation.^23^^,^^25^ Studies have shown that misconduct-related retractions are more frequent in countries with rapid research expansion and insufficient oversight.^28^ Furthermore, the persistence, even an increase, of postretraction citations suggests inadequate awareness and indexing of retracted work, risking the continued dissemination of erroneous findings. To address these issues, the scientific community must (1) prioritize quality research assessment over sheer publication volume, (2) enhance transparency through mandatory open data policies, and (3) strengthen editorial oversight with AI-based tools for fraud detection.^8^

The role of publishers in issuing retractions (73.0%) underscores the responsibility of journals to ensure the integrity of the published literature. This is in line with studies that argue that publishers play a critical role in detecting issues of misconduct, as they are often the first to be alerted to potential problems in published research.^33^ The fact that a minor proportion of retractions are initiated by authors (20.4%) or editors (23.2%) may reflect the reluctance of authors to self-report misconduct or errors, as well as the challenges editors face during the review process and in identifying quality issues postpublication.

The role of AI in scientific publishing warrants discussion in the context of these findings. On the one hand, AI and large language models (LLMs) are increasingly used in manuscript preparation, with evidence suggesting that researchers adopting LLM-based writing tools post significantly more papers, with increases of 36% to 60% depending on the field, with the largest gains among nonnative English speakers.^34^ However, this increase in output has not been matched by a proportional increase in scientific value, and the resulting flood of polished but potentially low-quality manuscripts places additional strain on the peer review system.^34^ On the other hand, AI-based tools are emerging as promising instruments for quality assurance and fraud detection. Automated screening tools such as the Problematic Paper Screener now routinely scan the published literature for textual fingerprints of paper mill activity.^35^ A recent machine learning model trained to identify paper mill products in cancer research flagged approximately 10% of all cancer publications from 1999 to 2024 as potentially fraudulent, with the problem most concentrated in molecular biology and early-stage laboratory research^36^—thematic areas that overlap substantially with the retracted studies identified in our analysis. Similarly, publisher-level integrity tools are being deployed to check author credentials, validate references, and detect AI-generated images and text prior to peer review.^35^ In this context, the high proportion of misconduct-related retractions observed in our study (77.8%) underscores the need for journals in radiation oncology to adopt such AI-assisted screening tools as part of their editorial workflows. At the same time, the growing use of LLMs by peer reviewers themselves—with estimates suggesting that between 7% and 17% of sentences in recent conference peer reviews were LLM-generated^37^—raises concerns about the depth and rigor of the review process. Thus, although AI holds considerable promise for strengthening research integrity, its uncritical adoption in both manuscript preparation and peer review may paradoxically contribute to the very quality issues it seeks to address.^38^

The high proportion of retracted studies marked with a watermark (93.5%) reflects an important shift toward greater transparency in the retraction process, especially as this figure has starkly increased in comparison to the one reported in the analysis conducted by Wasiak et al,^4^ where only 48% of retracted studies were marked with a watermark. This trend is consistent with broader calls for clearer communication regarding retractions in scientific publishing, especially given the advent of AI and chatbots.^39^ Interestingly, the median number of citations before retraction being 0 suggests that many of these studies were not widely cited prior to retraction, which may reflect their limited impact or the early detection of errors. However, the fact that the median number of citations after retraction is 1 indicates that at least some of these studies continued to be referenced, possibly due to lingering impact on the field or ongoing discussions regarding the retraction. A notable concern in postretraction citation patterns is the unintended increase in accessibility of retracted papers. As highlighted by Zietman et al,^40^ some retracted papers paradoxically become more widely available after their retraction because of the removal of paywalls and inconsistent database updates.

The finding that most retracted studies were published during and after the COVID-19 pandemic (72.2%), especially in the year 2022, is an important observation. During the COVID-19 pandemic, there was a rush to publish, which led to an increase in low-quality research, data errors, and even instances of scientific misconduct.^41^ The surge in publications during the pandemic has further increased pressure on researchers to publish, often under tight deadlines, which may have contributed to the higher incidence of retractions postpandemic, as researchers and publishers scrambled to ensure the reliability of studies.^42^ The median time to retraction (1.6 years) supports the idea that these issues were often identified relatively quickly but not always corrected promptly.

Based on these findings, the radiation oncology community can take away several key lessons: the high rate of misconduct-related retractions highlights the need for stronger ethical training and oversight, especially in fast-paced research settings. Researchers and institutions must prioritize rigorous methods, transparency, and ethical conduct to prevent retractions. The study also emphasizes the critical role of publishers in safeguarding research integrity and calls for improved misconduct detection. Lastly, the continued citation of retracted work underscores the importance of robust postpublication review to ensure scientific accuracy.

This systematic review's main strength lies in its comprehensive analysis of retracted publications in radiation oncology, highlighting trends such as the post–COVID-19 increase in retractions. However, its impact is limited by the small sample size (108 publications) and the lack of analysis of cultural or institutional factors—particularly relevant given the high proportion of studies from China. The limited data also restrict the study to descriptive trends, limiting deeper causal insights.

In conclusion, retractions in radiation oncology remain rare but significant. Misconduct remains the leading cause, with publishers playing a key role and notable geographic patterns emerging. The timing of retractions, especially around the COVID-19 pandemic, highlights the risks of rapid publishing. Future research should examine the long-term impact of these trends and ways to reduce misconduct in scientific publishing.

## Declaration of AI and AI-Assisted Technologies in the Writing Process

During the preparation of this work, the authors used chatGPT-4 from OpenAI in order to further improve the readibility and linguistic style of the manuscript. After using the tool, the authors reviewed and edited the content as needed and take full responsibility for the content of the publication.

## Disclosures

Felix Ehret has received honoraria and travel support from ZAP Surgical Systems, Inc and Accuray, Inc, and acknowledges research funding from the German Cancer Aid and Accuray, Inc, all unrelated to the submitted work. Daniel Zips received consulting or advisory fees, technical support, and research grants from Varian Medical Systems/Siemens Healthineers, Accuray, Therepanacea, and Sennewald, all unrelated to the submitted work. The other authors have no conflicts of interest to disclose.

## Appendix Supplementary materials

Supplements 1 Image, application 1

Supplements 2 Image, application 2

Supplements 3 Image, application 3

## Footnotes

Supplementary material associated with this article can be found in the online version at doi:10.1016/j.adro.2026.102071.

## References

[1] Van Noorden R. (2023). More than 10,000 research papers were retracted in 2023 — A new record. Nature.

[2] Fernandes B.B.P., Dodurgali M.R., Rossetti C.A., Pacheco-Barrios K., Fregni F. (2023). Editorial - The secret life of retractions in scientific publications. Princ Pract Clin Res.

[3] Xu C., Fan S., Tian Y. (2025). Investigating the impact of trial retractions on the healthcare evidence ecosystem (VITALITY Study I): Retrospective cohort study. BMJ.

[4] Wasiak J., Hamilton D.G., Foroudi F., Faggion C.M. (2018). Surveying retracted studies and notices within the field of radiation oncology. Int J Radiat Oncol Biol Phys.

[5] Hamilton D.G. (2019). Continued citation of retracted radiation oncology literature—Do we have a problem?. Int J Radiat Oncol Biol Phys.

[6] O’steen L., Indelicato D.J. (2018). Advances in the management of craniopharyngioma. F1000Res.

[7] Berger T., Noble D.J., Shelley L.E.A. (2021). 50 years of radiotherapy research: Evolution, trends and lessons for the future. Radiother Oncol.

[8] Catillon M. (2019). Trends and predictors of biomedical research quality, 1990–2015: A meta-research study. BMJ Open.

[9] Rooney M.K., Nesbit E.G., Holliday E.B. (2021). Trends in publication speed of radiation oncology research from 2010 to 2019. Adv Radiat Oncol.

[10] Wadhwa R.R., Rasendran C., Popovic Z.B., Nissen S.E., Desai M.Y. (2021). Temporal trends, characteristics, and citations of retracted articles in cardiovascular medicine. JAMA Netw Open.

[11] Qi Q., Huang J., Wu Y., Pan Y., Zhuang J., Yang X. (2024). Recent trends: Retractions of articles in the oncology field. Heliyon.

[12] Di Traglia R., Dunne H., Tysome J., Smith M.E. (2025). A systematic review of ENT retractions. Eur Arch Otorhinolaryngol.

[13] Huang A., Huang K.Y., Kim S.J. (2023). Retractions in dermatology literature between 1982 and 2022: Cross-sectional study. JMIR Dermatol.

[14] Kwee R.M., Kwee T.C. (2023). Retracted publications in medical imaging literature: An analysis using the retraction watch database. Acad Radiol.

[15] Baldock C., Basran P.S., Zaidi H. (2021). An increase in retractions of research publications is an issue for Medical Physics. Med Phys.

[16] Bolboacă S.D., Buhai D-V, Aluaș M., Bulboacă A.E. (2019). Post retraction citations among manuscripts reporting a radiology-imaging diagnostic method. PLoS One.

[17] Bratan T., Aichinger H., Brkic N. (2021). Impact of the COVID-19 pandemic on ongoing health research: An ad hoc survey among investigators in Germany. BMJ Open.

[18] Furuse Y. (2024). Characteristics of retracted research papers before and during the COVID-19 pandemic. Front Med (Lausanne).

[19] Page M.J., McKenzie J.E., Bossuyt P.M. (2021). The PRISMA 2020 statement: An updated guideline for reporting systematic reviews. BMJ.

[20] Christ S.M., Fritsak M., Kobeissi G. (2025). Navigating scientific progress in radiation oncology: Comprehensive analysis of clinical trials from the past two decades using the ClinicalTrials.gov database. JCO Glob Oncol.

[21] Hu X., Flahault A., Temerev A., Rozanova L. (2021). The progression of COVID-19 and the government response in China. Int J Environ Res Public Health.

[22] Conte M.L., Liu J., Schnell S., Omary M.B. (2017). Globalization and changing trends of biomedical research output. JCI Insight.

[23] Sebo P. (2024). Chinese authors are overrepresented in medical articles retracted for fake peer review or paper mill. Intern Emerg Med.

[24] Mallapaty S. (2024). China conducts first nationwide review of retractions and research misconduct. Nature.

[25] Fan J., Liu X., Li Y. (2022). Quality problems of clinical trials in China: Evidence from quality related studies. Trials.

[26] Wang F., Zhu C. (2023). Statistical analysis of research integrity construction in 466 Chinese universities with medical programs. Humanit Soc Sci Commun.

[27] The Lancet (2019). Research integrity: Time for global action. Lancet.

[28] Sebo P., Sebo M. (2025). Geographical disparities in research misconduct: Analyzing retraction patterns by country. J Med Internet Res.

[29] Corbyn Z. (2012). Misconduct is the main cause of life-sciences retractions. Nature.

[30] Stretton S., Bramich N.J., Keys J.R. (2012). Publication misconduct and plagiarism retractions: A systematic, retrospective study. Curr Med Res Opin.

[31] Nath S.B., Marcus S.C., Druss B.G. (2006). Retractions in the research literature: Misconduct or mistakes?. Med J Aust.

[32] Fang F.C., Steen R.G., Casadevall A. (2012). Misconduct accounts for the majority of retracted scientific publications. Proc Natl Acad Sci U S A.

[33] Schiff D. (2017). The importance of facts and the role of academic publishers in today’s world—A publisher’s view. Semin Respir Crit Care Med.

[34] Kusumegi K., Yang X., Ginsparg P., de Vaan M., Stuart T., Yin Y. (2025). Scientific production in the era of large language models. Science.

[35] Kocak Z. (2024). Publication ethics in the era of artificial intelligence. J Korean Med Sci.

[36] Scancar B., Byrne J.A., Causeur D., Barnett A.G. (2026). Machine learning based screening of potential paper mill publications in cancer research: Methodological and cross sectional study. BMJ.

[37] Liang W., Izzo Z., Zhang Y., Salakhutdinov R., Kolter Z., Heller K. (2024). Proceedings of the 41st International Conference on Machine Learning.

[38] Carobene A., Padoan A., Cabitza F., Banfi G., Plebani M. (2023). Rising adoption of artificial intelligence in scientific publishing: Evaluating the role, risks, and ethical implications in paper drafting and review process. Clin Chem Lab Med.

[39] Knight S., Viberg O., Mavrikis M. (2024). Emerging technologies and research ethics: Developing editorial policy using a scoping review and reference panel. PLoS One.

[40] Zietman A.L., Yom S.S., Braverman L.C. (2019). Making sure retractions matter. Int J Radiat Oncol Biol Phys.

[41] Sevryugina Y.V., Dicks A.J. (2022). Publication practices during the COVID-19 pandemic: Expedited publishing or simply an early bird effect?. Learn Publ.

[42] Suart C., Neuman K., Truant R. (2022). The impact of the COVID-19 pandemic on perceived publication pressure among academic researchers in Canada. PLoS One.

