# Supplementary material for: Integrity in Radiation Oncology Research: A Systematic Review of Retracted Studies, Retraction Notices, and Their Impact on the Field
Source: Adv Radiat Oncol. 2026 May 1;11(8):102071. doi: 10.1016/j.adro.2026.102071 (PMC13310937; doi:10.1016/j.adro.2026.102071)
Supplement: Supplements 2 [file mmc2.docx]

**Appendix E 2: GitHub Repository and Search Strategy**

**GitHub repository:** https://github.com/maksymfritsak/Preprocessing-for-Systematic-Review-of-Retracted-Studies

**Search String Pubmed/Medline and Cochrane Central:**

retracted AND (radiotherapy OR radiation OR irradiation OR radiooncology)

**Search String Embase:**

retracted AND ('radiotherapy'/exp OR radiotherapy OR 'radiation'/exp OR radiation OR 'irradiation'/exp OR irradiation OR radiooncology)
