# Supplementary material for: Integrity in Radiation Oncology Research: A Systematic Review of Retracted Studies, Retraction Notices, and Their Impact on the Field
Source: Adv Radiat Oncol. 2026 May 1;11(8):102071. doi: 10.1016/j.adro.2026.102071 (PMC13310937; doi:10.1016/j.adro.2026.102071)
Supplement: Supplements 3 [file mmc3.docx]

**Appendix E 3: List of assessed journal articles**

| **First author** | **Publication date** | **Journal** | **Country** | **Study Design** |
| --- | --- | --- | --- | --- |
| Ali Mohammad Alizadeh | 2020-06-01 | European Journal of Pharmacology | Iran | laboratory/experimental |
| Alizadeh, AM | 2020-05-05 | European Journal of Pharmacology | Iran | laboratory/experimental |
| Areumnuri Kim | 2021-09-03 | Cell Death & Disease | South Korea | laboratory/experimental |
| B G Fallone | 2016-04-06 | Physics in Medicine & Biology | Canada | laboratory/experimental |
| Baosheng Li | 2020-01-01 | American Journal of Cancer Research | China | laboratory/experimental |
| Berbeco I | 2017-07-12 | Nano Letters | USA | laboratory/experimental |
| Berbeco I | 2019-11-01 | Scientific reports | USA | laboratory/experimental |
| Cai, W | 2022-04-15 | Journal of Healthcare Engineering | China | retrospective observational |
| Chen, L | 2022-06-06 | Applied Bionics and Biomechanics | China | systematic review/metaanalysis |
| Chen, M | 2021-04-17 | Computational and Mathematical Methods in Medicine | China | retrospective observational |
| Chen, Q | 2021-11-25 | Journal of Healthcare Engineering | China | prospective observational |
| Chen, S | 2022-07-31 | Computational and Mathematical Methods in Medicine | China | prospective interventional |
| Chen, T | 2022-06-01 | Frontiers in Medicine | China | case report |
| Christos Papaloukas | 2010-07-21 | International Journal of General Medicine | Greece | case report |
| Chuanzhao Li | 2020-09-08 | Journal of Radiation Research | China | laboratory/experimental |
| Chunzi Gao | 2018-06-26 | Journal of Cellular Biochemistry | China | laboratory/experimental |
| Cleto S | 2020-11-01 | Leukemia research | Mexico | prospective interventional |
| Dai, Z | 2022-06-03 | Evidence-Based Complementary and Alternative Medicine | China | prospective randomised interventional |
| Ding, L | 2022-07-04 | Evidence-Based Complementary and Alternative Medicine | China | prospective observational |
| Dong Hong Zhang | 2018-11-08 | Cancer Management and Research | China | laboratory/experimental |
| Dong Wang | 2013-02-13 | PLoS One | China | laboratory/experimental |
| Feng, H | 2022-08-11 | Evidence-Based Complementary and Alternative Medicine | China | retrospective observational |
| Fengju Zhao | 2021-09-08 | Journal of Healthcare Engineering | China | laboratory/experimental |
| Fu, F | 2022-08-04 | Evidence-Based Complementary and Alternative Medicine | China | prospective randomised interventional |
| Gilda G Hillman | 2007-03-01 | Cancer Research | USA | laboratory/experimental |
| Gu, W | 2021-08-06 | Evidence-Based Complementary and Alternative Medicine | China | prospective observational |
| Gu, Z | 2022-09-29 | Contrast Media & Molecular Imaging | China | retrospective observational |
| Guangming Yi | 2020-01-06 | RSC Advances | China | laboratory/experimental |
| Guanyu Wang | 2021-11-27 | Oxidative Medicine and Cellular Longevity | China | laboratory/experimental |
| Haiyi Liu | 2022-09-28 | BioMed Research International | China | retrospective observational |
| Hong Yu | 2021-04-20 | BioMed Research International | China | prospective observational |
| Hridayesh Prakesh | 2019-02-12 | Frontiers in Immunology | India | laboratory/experimental |
| Huang N | 2022-03-21 | Evidence-Based Complementary and Alternative Medicine | China | prospective interventional |
| Isabella M Grumbach | 2019-11-09 | Free Radical Biology & Medicine | USA | laboratory/experimental |
| Isik N | 2022-01-01 | Journal of Cancer Research and Therapeutics | Turkey | retrospective observational |
| Isik N | 2023-04-01 | Journal of Cancer Research and Therapeutics | Turkey | retrospective observational |
| Jasti S Rao | 2011-03-29 | International Journal of Oncology | USA | laboratory/experimental |
| Jasti S Rao | 2010-12-10 | Molecular Cancer Research (MCR) | USA | laboratory/experimental |
| Jasti S Rao | 2011-06-16 | PLoS One | USA | laboratory/experimental |
| Jianrong Zhou | 2021-09-28 | Pharmacogenomics and Personalised Medicine | China | retrospective observational |
| Jin, H | 2022-04-25 | Disease Markers | China | retrospective observational |
| Lakosha, H | 2017-05-01 | Oman Journal of Ophthalmology | Canada | case report |
| Li H | 2022-06-17 | Applied Bionics and Biomechanics | China | prospective interventional |
| Li J | 2022-02-15 | Journal of Healthcare Engineering | China | prospective interventional |
| Li M | 2022-09-23 | Contrast Media & Molecular Imaging | China | retrospective observational |
| Li Ping Peng | 2017-10-09 | Cellular Physiology and Biochemistry | China | laboratory/experimental |
| Li X | 2022-09-23 | Evidence-Based Complementary and Alternative Medicine | China | prospective interventional |
| Li, D | 2022-08-09 | Contrast Media & Molecular Imaging | China | prospective interventional |
| Li, H | 2022-09-23 | Contrast Media & Molecular Imaging | China | retrospective observational |
| Li, X | 2022-06-25 | BioMed Research International | China | retrospective observational |
| Li, X | 2022-09-23 | Evidence-Based Complementary and Alternative Medicine | China | retrospective observational |
| Lin Z | 2022-09-24 | Evidence-Based Complementary and Alternative Medicine | China | retrospective observational |
| Lin, RS | 2022-03-12 | Journal of Healthcare Engineering | China | retrospective observational |
| Liu, F | 2021-10-13 | Journal of Healthcare Engineering | China | retrospective observational |
| Lu, Y | 2022-07-13 | Computational and Mathematical Methods in Medicine | China | prospective observational |
| Luo, Z | 2020-10-30 | Technology in Cancer Research & Treatment | China | laboratory/experimental |
| Ma J | 2022-03-08 | Journal of Healthcare Engineering | China | prospective interventional |
| Ma, Y | 2022-05-04 | Computational Intelligence and Neuroscience | China | retrospective observational |
| Malone S | 2021-12-28 | Cureus | Canada | case report |
| Mao, X | 2022-02-02 | Journal of Healthcare Engineering | China | retrospective observational |
| Mohsen Shoja | 2019-08-19 | IET Nanobiotechnology | Iran | laboratory/experimental |
| Muxiang Zhou | 2015-03-24 | Molecular Oncology | USA | laboratory/experimental |
| Nassar Al-Rajhi | 2013-05-22 | Radiation Oncology | Saudi Arabia | prospective observational |
| Neto, CP | 2023-11-10 | Journal of Neurosurgery | USA | retrospective observational |
| Ping Hu | 2020-06-15 | European Review for Medical and Pharmacological Sciences | China | laboratory/experimental |
| Pistevou-Gompaki K | 2009-07-30 | International Journal of General Medicine | Greece | case report |
| Prophylactic Cranial Irradiation Overview Collaborative Group | 2000-10-23 | Cochrane Database of Systematic Reviews | France | systematic review/metaanalysis |
| Qian, X | 2021-11-19 | Journal of Healthcare Engineering | China | laboratory/experimental |
| Qiang Liu | 2018-07-17 | Cellular Physiology and Biochemistry | China | laboratory/experimental |
| Qin Zhou | 2022-03-02 | Journal of Healthcare Engineering | China | laboratory/experimental |
| Qirong Li | 2018-08-01 | European Review for Medical and Pharmacological Sciences | China | laboratory/experimental |
| Rosella Galati | 2019-10-13 | Therapeutic Advances in Medical Oncology | Italy | laboratory/experimental |
| Rutong Yu | 2020-03-30 | Cancer Cell International | China | laboratory/experimental |
| Saeeda Saeeda | 2021-10-02 | Molecules | Bangladesh | systematic review/metaanalysis |
| Sam S Yoon | 2013-11-01 | Molecular Cancer Therapeutics | South Korea | laboratory/experimental |
| Shan Qing Li | 2016-11-30 | Tumor Biology (Tumour Biology) - Official Journal of the International Society of Oncology and BioMarkers (ISOBM) | China | laboratory/experimental |
| Silva L | 2018-01-17 | Hematology | Mexico | prospective interventional |
| Soo Ok Lee | 2019-06-11 | Journal of Molecular Medicine: continuation of Klinische Wochenschrift | China | laboratory/experimental |
| Soo Ok Lee | 2015-11-14 | Radiation Oncology | USA | laboratory/experimental |
| Tang Y | 2022-06-14 | Evidence-Based Complementary and Alternative Medicine | China | prospective interventional |
| Teimourian S | 2018-09-04 | Medical physics | Iran | prospective observational |
| Tian L | 2020-03-01 | Oral surgery, oral medicine, oral pathology and oral radiology | China | systematic review/metaanalysis |
| Wang C | 2022-06-28 | Computational and Mathematical Methods in Medicine | China | retrospective observational |
| Wang H | 2022-04-05 | Journal of Healthcare Engineering | China | retrospective observational |
| Wang W | 2022-04-11 | Journal of Oncology | China | retrospective observational |
| Wang X | 2022-07-28 | Computational and Mathematical Methods in Medicine | China | prospective observational |
| Wang, N | 2023-06-30 | Medicine | China | retrospective observational |
| Wei D | 2022-08-24 | Computational and Mathematical Methods in Medicine | China | systematic review/metaanalysis |
| Wei Min Zhang | 2018-03-26 | Journal of Cancer Research and Therapeutics | China | retrospective observational |
| Wenwen Wei | 2019-06-10 | Bioscience Reports | China | laboratory/experimental |
| Wu G | 2022-07-11 | Computational and Mathematical Methods in Medicine | China | retrospective observational |
| Wu, C | 2023-04-13 | Evidence-Based Complementary and Alternative Medicine | China | prospective randomised interventional |
| Xian Hou Yuan | 2007-09-04 | World Neurosurgery | China | laboratory/experimental |
| Xiao Rong Dong | 2020-10-29 | Therapeutic Advances in Medical Oncology | China | laboratory/experimental |
| Xiaosong Gu | 2020-09-14 | Cancer Management and Research | China | laboratory/experimental |
| Ya Wang | 2004-01-16 | Nucleic Acids Research | Germany | laboratory/experimental |
| Ya Wang | 2014-09-10 | The Journal of Biological Chemistry | USA | laboratory/experimental |
| Ye Min Liang | 2017-03-23 | OncoTargets and Therapy | China | laboratory/experimental |
| Yin R | 2021-11-12 | Journal of Healthcare Engineering | China | retrospective observational |
| Yinglong Hou | 2021-12-31 | Computational and Mathematical Methods in Medicine | China | retrospective observational |
| Yiyuan Song | 2022-03-18 | Journal of Healthcare Engineering | China | systematic review/metaanalysis |
| Zakariae Benjelloun | 2018-12-04 | Journal of Radiotherapy in Practice | Morocco | prospective observational |
| Zhang H | 2021-08-22 | Lancet Oncology | China | prospective interventional |
| Zhang Y | 2018-02-22 | Cancer Imaging | China | prospective observational |
| Zhang Y | 2022-07-14 | Contrast Media & Molecular Imaging | China | retrospective observational |
| Zhang, L | 2021-08-17 | Journal of Healthcare Engineering | China | prospective observational |
| Zhong Ye | 2022-05-31 | Applied Bionics and Biomechanics | China | retrospective observational |
| Zilong Chen | 2019-11-24 | Cell Cycle | China | laboratory/experimental |
